# Eating Disorder Symptoms and Proneness in Gay Men, Lesbian Women, and Transgender and Gender Non-conforming Adults: Comparative Levels and a Proposed Mediational Model

**DOI:** 10.3389/fpsyg.2018.02692

**Published:** 2019-01-08

**Authors:** Kathryn Bell, Elizabeth Rieger, Jameson K. Hirsch

**Affiliations:** ^1^Research School of Psychology, Australian National University, Canberra, ACT, Australia; ^2^Department of Psychology, East Tennessee State University, Johnson City, TN, United States

**Keywords:** eating disorders, sexual minority and gender diverse, self-compassion, stigma, interpersonal, LGBTQ

## Abstract

In this study we sought to compare eating disorder attitudes and behaviors, and proneness to an eating disorder (“ED proneness”), between gay men, lesbian women, and transgender and gender non-conforming (TGNC) adults. A further aim was to identify and compare risk and protective factors, and examine a mediational model based on the interpersonal theory of eating disorders (IPT-ED), whereby the association between interpersonal factors and ED proneness would be mediated by psychological constructs pertaining to the self and negative affect. Data was obtained from a larger national study of health risk and protective factors among sexual minority and gender diverse populations. The sample included 97 gay men, 82 lesbian women, and 138 TGNC adults. Participants completed the National College Health Assessment, Eating Disorders Screen for Primary Care, Patient Health Questionnaire Depression scale, Generalized Anxiety Disorder 7 scale, Self-Compassion Scale-Short Form, Negative Social Exchange subscale of the Multidimensional Health Profile, Interpersonal Needs Questionnaire, and Perceived Stigma Scale. There was a significant difference between groups in ED proneness, with lesbian women (66.7%) having a significantly higher percentage than gay men (47.6%). There was also a significant difference between groups in weight-based self-worth, with the lowest percentage in gay men (63%) and the highest percentage in lesbian women (82%), as well as dissatisfaction with eating patterns, with the highest percentage in TGNC adults (69.8%) and the lowest percentage in gay men (47.7%). There was a low percentage of inappropriate compensatory behaviors, with no significant difference between groups. Logistic regression analyses showed that the predictor variables of ED proneness were depression, perceived stigma, and self-compassion in gay men; depression in lesbian women; and self-compassion in the TGNC adults. Mediation analyses showed that thwarted belongingness (i.e., an unmet to belong) and perceived stigma had an indirect association with ED proneness that was mediated by self-compassion and depression (for perceived stigma alone) in gay men, depression in lesbian women, and self-compassion in TGNC adults. The interpersonal theory of eating disorders therefore extends to sexual minority and gender diverse populations; however, the results suggest a broadening of theoretical models and intervention programs to include the role of stigma and self-compassion.

## Introduction

Eating disorders are serious conditions “characterized by a persistent disturbance of eating or eating-related behavior that results in the altered consumption or absorption of food and that significantly impairs physical health or psychosocial functioning” (American Psychiatric Association, 2013, p. 329). A core diagnostic feature of most eating disorders is that concerns about weight and shape unduly influence a person's self-worth (American Psychiatric Association, 2013). Behavioral features of eating disorders can include extreme dieting, binge eating episodes, and inappropriate compensatory behaviors such as self-induced vomiting, laxative or diuretic misuse, misuse of other medication (e.g., diet pills), fasting, or excessive exercise (American Psychiatric Association, 2013). A review of epidemiological studies reported a lifetime prevalence of approximately one percent for anorexia nervosa and two percent for bulimia nervosa among women, and an estimated lifetime prevalence of two percent for binge-eating disorder across males and females (Smink et al., 2013). Prevalence rates, however, are much higher in terms of subclinical eating disorders (e.g., 23% of women were defined as currently experiencing clinically significant levels of eating disordered attitudes and behaviors in one study) (Wade et al., 2012). Five-year recovery percentages for eating disorders have been reported as 40–50% for anorexia nervosa, depending on how recovery was defined in the study, with ~20% developing a chronic course, and 55% for bulimia nervosa, underscoring the chronicity of these disorders for many individuals (Steinhausen, 2002; van Son et al., 2010; Smink et al., 2013).

Given these concerning data in terms of prevalence and recovery rates, research on eating disorder pathology has sought to identify risk and protective factors to enable prevention and early intervention and thereby reduce chronicity. Among the most common risk factors that have been identified to date are shape and weight concerns, internalization of the thin ideal, social pressure to be thin, dietary restraint, a family history of disordered eating, perfectionism, childhood teasing, negative affect, and impulsivity (particularly for binge-eating disorder) (Stice, 2002; Striegel-Moore and Bulik, 2007; Stice et al., 2011; Hilbert et al., 2014). While protective factors remain relatively underexplored, social support has been found to be one such factor in eating disorder pathology (Stice, 2002; Hilbert et al., 2014; Mason et al., 2017; Watson et al., 2017), and emerging evidence suggests that self-compassion may also be relevant in this regard (Braun et al., 2016).

These risk and protective factors variously feature in the diverse theoretical models that have been advanced to account for the development and/or maintenance of eating disorder symptoms (Pennesi and Wade, 2016). Rieger et al. (2010) proposed an interpersonal theory of eating disorders (IPT-ED) that integrates interpersonal and psychological difficulties in understanding the emergence of eating disorder symptoms. This model highlights the role of inadequate social interactions, defined as those that share the core feature of entailing real or perceived negative evaluation by others, such as thwarted belongingness (i.e., an unmet need to belong) or negative social exchanges (Ruehlman et al., 1999; Van Orden et al., 2012). These inadequate social interactions are theorized to lead to disturbances of the self, including low self-esteem and negative affect, which in turn trigger and perpetuate eating disorder symptoms (e.g., dieting to enhance self-esteem and binge eating to regulate negative affect). Models of this kind can be used to inform investigations of risk and protective factors, and their interaction, for eating disorder symptoms.

Among current theoretical conceptualizations of eating disorders, the IPT-ED model might be especially applicable to those populations susceptible to experiencing negative social evaluation such as sexual minority and gender diverse populations. McClain and Peebles (2016) note that much of the research into eating disorders has been conducted on the female, heterosexual, and cisgender population, such that research into sexual minority and gender diverse populations is needed. The limited research that has been conducted to date suggests that sexual orientation and gender identity have a distinct association with eating disorder pathology (Feldman and Meyer, 2007). For example, in a population study of youth, while the majority had positive body image and did not develop eating concerns, gay and bisexual secondary school boys and girls were each more likely to report purging and diet pill use than their heterosexual counterparts (Austin et al., 2013). This finding of elevated risk extends to adults, with research indicating that gay men are at greater risk than heterosexual men for developing eating disorders (Striegel-Moore and Bulik, 2007; Feldman and Meyer, 2010) and have a higher incidence of drive for thinness, body dissatisfaction, and body image related anxiety (McClain and Peebles, 2016). There is mixed data on the prevalence of eating disorder symptoms in lesbian women, with some studies suggesting an increased prevalence of binge eating behaviors relative to heterosexual women (Feldman and Meyer, 2010; Bayer et al., 2017).

Even less research has been conducted on transgender and gender non-conforming (TGNC) people (American Psychiatric Association, 2015). A large-scale study of college students by Diemer et al. (2015) found that the prevalence of self-reported disordered eating and inappropriate compensatory behavior was higher among transgender students than cisgender gay or heterosexual students. McClain and Peebles (2016) explain these findings by noting that some TGNC people experience dissatisfaction or distress in relation to features of their biological sex that are inconsistent with their gender identity, and propose that for these individuals the risk of eating disorder symptoms may be secondary to this body dissatisfaction or distress. For example, extreme weight loss strategies among TGNC people may be a means to inhibit undesired, or develop desired, gender features (Diemer et al., 2015; Watson et al., 2017). If this is the case, eating disorder symptoms in this group might be best alleviated with strategies to ameliorate this body dissatisfaction, including social transition, puberty blockers, cross-sex hormones, and gender affirmation surgery compared to implementing standard treatment approaches for eating disorders. In accordance with this suggestion, a systematic review of body dissatisfaction and disordered eating in TGNC people by Jones et al. (2016) found that body image improved after hormone or surgical treatments targeting dissatisfaction with the physical characteristics of one's biological sex that were discrepant from one's gender identity.

In summary, much of the previous research on eating disorders has investigated risk and protective factors within a particular group (primarily heterosexual and cisgender girls and women) and has failed to consider sexual minority and gender diverse groups, or has generalized across diverse sexual minority and gender diverse populations. Moreover, research that has addressed eating disorders in sexual minority and gender diverse sub-groups has focused primarily on the prevalence of eating disorder symptoms rather than investigating risk and protective factors in sexual minority and gender diverse sub-groups. This neglect is noteworthy since it cannot be assumed that the same etiological factors, and hence intervention approaches, are relevant across diverse groups. Indeed, as previously highlighted, it has been proposed that mainstream theoretical and intervention approaches may not be relevant for those TGNC people for whom eating disorder symptoms might be primarily driven by dissatisfaction with sex features that are inconsistent with one's gender identity and that are, therefore, ameliorated through interventions that target this dissatisfaction.

The need for examining novel constructs in understanding the emergence of eating disorder symptoms among sexual minority and gender diverse groups is underscored by research into the determinants of health within this population, which notes that this community faces additional and unique stressors, such as stigma, that impact health outcomes (Kelleher, 2009; Hatzenbuehler and Pachankis, 2016). Mickelson (2001) posits that perceived stigma (i.e., a person's perception of stigma toward them) has a greater impact on social support and depression than the actual stigma toward an individual. Perceived stigma has been shown to account for increased depression in sexual minority and gender diverse populations (Lewis et al., 2003; Almeida et al., 2009). Wang and Borders (2017) found that perceived discrimination was a unique correlate of eating disorder symptoms in a sample of 116 sexual minority individuals. A comparable relationship was observed by Watson et al. (2017) whereby the risk of eating disorder pathology in transgender youth was related to stigma, and this risk was reduced by higher levels of perceived social support. Similarly, Mason et al. (2017) found that among lesbian young women, sexual orientation discrimination had an indirect role on eating disorder symptoms. More specifically, sexual orientation discrimination was related to less social support from family which, in turn, was related to increased negative affect and social anxiety. Negative affect and social anxiety were, in turn, directly related to eating disorder symptoms. These findings suggest that, consistent with the IPT-ED model, perceived stigma (a form of negative social evaluation) may increase negative affect, thereby increasing the risk of eating disorder symptoms as a means of regulating affect. As such, the IPT-ED model can be seen as an eating disorder specific instantiation of Meyer's (2003) minority stress model which posits that, “stigma, prejudice, and discrimination create a hostile and stressful social environment that causes mental health problems” in minority groups such as gay, lesbian, and TGNC populations (p. 674).

While perceived stigma may be an eating disorder risk factor that is particularly relevant to sexual minority and gender diverse populations, self-compassion may be uniquely protective in this group as it may help to protect against the negative effects of stigmatizing experiences. Neff (2003) defines self-compassion as encompassing three elements: self-kindness [i.e., “extending kindness and understanding to oneself rather than harsh judgment and self-criticism” (p. 89)], common humanity [i.e., “seeing one's experiences as part of the larger human experience rather than seeing them as separating and isolating” (p. 89)], and mindfulness [i.e., “holding one's painful thoughts and feelings in balanced awareness rather than over-identifying with them” (p. 89)]. Previous research has found that self-compassion mediates the association between bias-based bullying and mental health symptoms in sexual minority youth (Vigna et al., 2017) and self-compassion is positively related to well-being in gay men and TGNC adults (Beard et al., 2017; Keng and Liew, 2017). More broadly, self-compassion appears to serve as a protective factor in mental health outcomes. Neff (2003) suggests that self-compassion plays an adaptive role in emotion regulation, thereby promoting beneficial health outcomes. Consistent with this, in a meta-analysis by MacBeth and Gumley (2012), a large effect size was found for the inverse association between self-compassion and psychopathology. While this meta-analysis focused on depression, anxiety, and stress, it provides a rationale for exploring the association of self-compassion with eating disorder symptoms, as studies have begun to do (Braun et al., 2016). For example, Geller et al. (2015) found that self-compassion accounted for unique variance in shape and weight concerns and eating disorder symptoms in a community sample of women. Similarly, Tylka et al. (2015) found that women who were higher in self-compassion perceived lower thinness-related pressure, particularly from the media. The role of self-compassion has also been supported in clinical samples of people with eating disorders. For instance, a study by Kelly and Tasca (2016) explored the relationship between self-compassion, shame, and eating disorder symptoms among patients with eating disorders over the course of day hospital or inpatient treatment. It was found that, over the course of treatment, patients who had higher levels of self-criticism (or were low in self-compassion) had higher levels of shame and more severe eating disorder symptoms. Overall, the findings linking self-compassion and eating disorder symptoms can be understood in terms of the IPT-ED model: while negative social evaluation would be expected to trigger self-criticism (reduced self-compassion) overall, individuals will be protected from eating disorder pathology to the degree to which they can respond to this negative evaluation in a self-compassionate manner. This self-compassionate response provides a means of countering the potentially adverse effects of negative social evaluation on self-esteem and mood that leave the individual vulnerable to eating disorder symptoms.

In light of the aforementioned considerations, the rationale for our study was to (1) contribute to the minimal research to date that has compared eating disorder symptoms in gay men, lesbian women, and TGNC adults; (2) understand the factors that may be involved in susceptibility to an eating disorder in these groups using an empirically-supported theoretical model (i.e., IPT-ED) that has particular relevance to populations vulnerable to experiencing negative social evaluation; and (3) move beyond assessing the correlates of eating disorder pathology to understand how these variables interact with one another through our test of a meditational model based on the IPT-ED theory.

More specifically, in the present study, we investigated and compared the percentage of eating disorder symptoms (including maladaptive weight control behaviors, dissatisfaction with eating patterns, eating in secret, and weight-based self-worth) and proneness to an eating disorder (“ED proneness”) in gay cisgender men (gay men), lesbian cisgender women (lesbian women), and TGNC adult populations. While only limited research has been undertaken, available data suggests comparable prevalence rates in eating disorders between gay men and lesbian women (Feldman and Meyer, 2007) but that TGNC individuals have higher levels of eating disorder symptoms compared to gay men and lesbian women (Diemer et al., 2015). Hence it was hypothesized that TGNC adults would have the highest levels of eating disorder symptoms and ED proneness, with no difference in this regard between the gay men and lesbian women.

A further aim of the study was to investigate and compare psychological (i.e., depression, anxiety, and self-compassion) and interpersonal (i.e., perceived stigma, negative social exchange, and thwarted belongingness) risk and protective factors of ED proneness between these groups. We believed it was important to investigate risk and protective factors of ED proneness, as opposed to diagnosed eating disorders, given longitudinal research indicating that, “even apparently minor levels of [eating disorder] symptomatology are associated with significant and far-reaching deficits in well-being” (Wade et al., 2012, p. 352). In accordance with the IPT-ED model, we hypothesized that negative social evaluation, as measured by thwarted belongingness, negative social exchange, and perceived stigma, would have an indirect association with ED proneness that is mediated by negative affect and (lower) self-compassion. Finally, since research supports the notion that eating disorder pathology among the transgender population is largely or entirely secondary to dissatisfaction with sex features that are inconsistent with one's gender identity, we hypothesized that the risk and protective factors of ED proneness in this group would differ from those of cisgender gay men and lesbian women.

## Materials and Methods

### Participants

Participants in the present study (*n* = 267) were recruited as part of a national study (*N* = 496) conducted by the Laboratory for Resilience in Psychological and Physical Health in Johnson City, USA, investigating risk and protective factors for mental and physical health in sexual minority and gender diverse populations (Hirsch et al., 2017). Ethical permission was obtained from the Institutional Review Board of East Tennessee State University.

In the current study, those who identified their birth sex and gender identity as male, and sexual orientation as gay were grouped as “gay men.” Those who identified their birth sex and gender identity as female, and sexual orientation as lesbian were grouped as “lesbian women.” Participants who indicated that their gender identity was different from their birth sex (*n* = 26) or who identified as transgender male to female (*n* = 14), transgender female to male (*n* = 21), gender queer (*n* = 21), gender-fluid (*n* = 8), non-binary (*n* = 17), agender (*n* = 6), two-spirit (*n* = 5), intersex (*n* = 2), or other (*n* = 18) were collectively grouped as “TGNC” so as to maximize power. The final sample was comprised of 98 gay men (mean age = 42.54, *SD* = 17.19; 74.5% Caucasian), 84 lesbian women (mean age = 38.64, *SD* = 17.65; 84.5% Caucasian), and 138 TGNC adults (mean age = 33.60, *SD* = 16.80; 83.3% Caucasian). Within the TGNC group, 46 identified their sex assigned at birth as male, 89 as female, and three declined to answer. Demographic characteristics of the three groups are shown in Table 1.

**Table 1:** Participant demographic characteristics in percentages.

	**Gay men (*n* = 98)**	**Lesbian women (*n* = 84)**	**TGNC adults (*n* = 138)**
Mean Age (SD)	42.54 (17.19)	38.64 (17.65)	33.60 (16.80)
**LEGAL MARITAL STATUS**			
Single, never married	49.0	50.0	48.6
Married	20.4	21.4	21.7
Divorced	7.1	2.4	21.7
Widowed	1.0	0.0	0.0
Partnered, not legally married	15.3	21.4	16.7
Other	4.1	4.8	1.4
Decline to answer	1.0	0.0	1.4
Missing response	2.0	0.0	0.0
**HIGHEST EDUCATION LEVEL**			
Some high school	0.0	0.0	3.6
High school (includes GED)	4.1	3.6	3.6
Some college (no degree)	19.4	26.2	34.1
Associate's degree (2 years of college)	3.1	10.7	7.2
Bachelor degree (4 years of college)	33.7	21.4	23.9
Masters degree	27.6	23.8	19.6
Doctoral degree	12.2	14.3	7.2
Missing response	0.0	0.0	0.7
**EMPLOYMENT STATUS**			
Full time, paid	54.1	41.7	40.6
Part time, paid	5.1	10.7	12.3
Student	15.3	28.6	26.1
Homemaker	0.0	0.0	0.7
Retired	19.4	6.0	4.3
On disability	0.0	6.0	3.6
Unemployed, seeking paid employment	1.0	0.0	4.3
Unemployed, not seeking paid employment	0.0	1.2	0.7
Other	5.1	6.0	7.2
**ETHNICITY**			
White/Caucasian	74.5	84.5	83.3
Black or African American	2.0	1.2	2.2
Hispanic or Latino/a	9.2	0.0	1.4
American Indian or Alaska Native	0.0	1.2	0.7
Asian (including Asian Indian)	4.1	3.6	0.7
Multiracial	3.1	7.1	5.8
Other	7.1	0.0	4.3
Decline to answer	0.0	0.0	1.4
Missing data	0.0	1.2	0.0

### Measures

A demographics questionnaire was administered that asked participants to identify their age, sex at birth (male, female), gender identity (male, female, transgender male to female, transgender female to male, gender queer, gender-fluid, non-binary, agender, two-spirit, intersex, or other), ethnicity, and sexual orientation (heterosexual/straight, gay, lesbian, bisexual, pansexual, asexual, queer, or other). In addition, the following measures were administered.

#### National College Health Assessment (NCHA; American College Health Association, 2014)

The weight management item from the NCHA was included to assess maladaptive weight control behaviors. Respondents are asked to answer “yes” or “no” as to whether they have engaged in the following behaviors in the last 30 days: exercised, dieted, vomited/taken laxatives, or taken diet pills to lose weight. Participants were able to endorse as many of the items as were relevant. Exercise and dieting to lose weight were not included in this study as there was no way to determine if these behaviors were disordered by being excessive or inappropriate.

#### Eating Disorders Screen for Primary Care (ESP; Cotton et al., 2003)

The ESP is a five-item screening instrument for eating disorders. Respondents answer “yes” or “no” to the items: “Are you satisfied with your eating patterns?” “Do you ever eat in secret,” “Does your weight affect the way you feel about yourself?” “Have any members of your family suffered with an eating disorders?” and “Do you currently suffer with or have you ever suffered in the past with an eating disorder?” The item assessing satisfaction with eating patterns was reverse scored to index dissatisfaction. To determine ED proneness, we used a cut-off score of two, and excluded the item, “Have any members of your family suffered with an eating disorder?” as recommended by Cotton et al. (2003). This cut-off score has been found to yield a sensitivity of 100% and specificity of 71% in the identification of an eating disorder. We chose to emphasize sensitivity over specificity since even sub-clinical eating disorder presentations have been found to predict marked deteriorations in quality of life over time (Wade et al., 2012). However, given the lower specificity of the ESP, and thus the tendency to falsely diagnose some individuals who do not have an eating disorder as having one, we refer to this measure as indexing ED proneness rather than an actual eating disorder. In comparison to the SCOFF, another screening instrument for eating disorders, the ESP has the advantages of greater sensitivity (100% for the ESP vs. 78% for the SCOFF) and including an item assessing the core cognitive psychopathology of eating disorders (in terms of both diagnosis and as a maintenance factor), namely, weight-based self-worth (Fairburn et al., 2003).

#### Patient Health Questionnaire Depression Scale (PHQ-9; Kroenke et al., 2001)

The PHQ-9 is a nine-item self-report measure of depression over the previous 2 week period. Items align with the *DSM-IV* diagnostic criteria of depression (e.g., “feeling down, depressed, or hopeless” and “feeling tired or having little energy”) and are rated on a four-point Likert scale ranging from 0 (*not at all*) to 3 (*nearly every day*). The PHQ-9 has demonstrated high internal consistency (Cronbach's α = 0.86–0.89) and construct validity (Kroenke et al., 2001; Martin et al., 2006). Cronbach's alphas for each group were 0.86 in gay men, 0.90 in lesbian women, and 0.93 in TGNC adults.

#### Generalized Anxiety Disorder 7 (GAD-7; Spitzer et al., 2006)

The GAD-7 is a seven-item self-report measure that assesses the frequency of GAD symptoms over the preceding 2-week period. The items align with *DSM-IV* criteria for GAD (e.g., “feeling nervous, anxious or on edge” and “worrying too much about different things”). Responses are rated on a four-point Likert scale ranging from 0 (*not at all*) to 3 (*nearly every day*). The scale has demonstrated high internal consistency (Cronbach's α = 0.92) and test-retest reliability (*r* = 0.83) (Spitzer et al., 2006). A systematic review found that the GAD-7 was the best performing test in the assessment of GAD (Herr et al., 2014). Cronbach's alphas for each group were 0.92 in gay men, 0.93 in lesbian women, and 0.90 in TGNC adults.

#### Self-Compassion Scale-Short Form (SCS; Raes et al., 2011)

The SCS is a 12-item self-report scale that asks respondents to percentage on a five-point Likert scale (1 = *almost never*, 5 = *almost always*) how they typically act toward themselves in difficult times (e.g., “When I'm going through a hard time, I give myself the caring and tenderness I need”). The scale demonstrated good internal consistency and is highly correlated with the long version (Raes et al., 2011). Cronbach's alphas for each group were 0.90 in gay men, 0.91 in lesbian women, and 0.89 in TGNC adults.

#### Negative Social Exchange Subscale of the Multidimensional Health Profile: Psychological Functioning (MHP-P; Ruehlman et al., 1999)

Negative social interactions were measured by the four-item Negative Social Exchange subscale of the MHP-P. Items such as “Over the past year, how often were your close friends or close family angry, hostile, or impatient with you” are scored on a five-point Likert scale (1 = *never*, 5 = *very often*). This subscale has demonstrated good test-retest reliability (*r* = 0.77) as well as construct validity (Ruehlman et al., 1999). Cronbach's alphas for each group were 0.83 in gay men, 0.91 in lesbian women, and 0.85 in TGNC adults.

#### Interpersonal Needs Questionnaire (INQ; Van Orden et al., 2012)

The INQ is a 15-item self-report scale that includes the subscales of Thwarted Belongingness (nine items) and Perceived Burdensomeness (six items). Items are rated on a seven-point Likert scale ranging from 1 (*not at all true for me*) to 7 (*very true for me*). Only items from the Thwarted Belongingness subscale, which measures an unmet need to belong (e.g., “These days, other people care about me,” reverse scored), were used in the study, as this subscale alone has demonstrated a significant correlation with eating disorder symptoms (Silva et al., 2015). Positive items are reverse scored, such that a higher score indicates higher thwarted belongingness. Cronbach's alphas for each group on the Thwarted Belongingness subscale were 0.91 in gay men, 0.91 in lesbian women, and 0.90 in TGNC adults.

#### Perceived Stigma Scale (PSS; Mickelson, 2001)

The PSS is an eight-item self-report questionnaire that was adapted from a scale assessing perceived stigma among parents of children with special needs (Mickelson, 2001). The adapted items for this study ask respondents to rate their own, and their perception of other people's, attitudes and feelings about the respondent's sexual orientation on a five-point Likert scale from 1 (*definitely disagree*) to 5 (*definitely agree*). Example items include, “People have treated me differently because of my sexual orientation” and “I have felt odd/abnormal” because of my sexual orientation.” The original scale has demonstrated adequate internal consistency and test-retest reliability (Mickelson, 2001). Cronbach's alphas for each group were 0.86 in gay men, 0.81 in lesbian women, and 0.77 in TGNC adults.

### Procedure

The research was advertised as a study investigating resilience and health-related quality of life in the LGBTQ community. Invitations were extended via support organizations and social media; for example, recruitment information was sent to local, regional, and national peer-support groups, community helping agencies, and national advocacy organizations (Hirsch et al., 2017). Participants self-selected to take part in the study and completed each of the measures online using SurveyMonkey. All participants provided electronic informed consent, which required them to acknowledge their understanding of the procedures of the study, including risks and benefits, prior to administration of the survey materials. Participation was voluntary and there was no remuneration or compensation for taking part in the study. Participants were provided with the details of mental health resources when they completed the survey.

### Statistical Analysis

Analysis was conducted using SPSS-version 22. There was a significant main effect for age [*F*_(2, 314)_ = 8.88*, p* < 0.001, *d* = −0.43], with Tukey HSD *post-hoc* analysis indicating that gay men were significantly older than TGNC adults (*p* < 0.001). As such, age was controlled for in all analyses that compared groups. ED proneness was coded as a dichotomous variable where an ESP score of two or more represented ED proneness (Cotton et al., 2003). Logistic regressions were conducted to calculate odd ratios (*OR*) and 95% confidence intervals (CI) to examine whether there were significant differences between groups for the dichotomous variables, namely, percentage of eating disorder symptoms as measured by the presence of inappropriate compensatory behaviors, dissatisfaction with eating patterns, current/past eating disorder, eating in secret, and weight-based self-worth, as well as by ED proneness, while controlling for age. These were conducted firstly with gay men, and then again with TGNC adults as the reference groups to enable all significant differences to be detected. One-way analyses of variance (ANOVAs) were conducted to compare the means for each predictor variable (depression, anxiety, self-compassion, negative social exchange, thwarted belongingness, and perceived stigma) between groups, with *post-hoc* comparisons using the Tukey HSD test. Pearson correlations were performed for each predictor variable (depression, anxiety, self-compassion, negative social exchange, thwarted belongingness, and perceived stigma) to check for multicollinearity, of which there was none. Logistic regressions were conducted to identify which variables were significant predictor variables for ED proneness for each group. Based on the results of the correlations and logistic regressions, mediation analyses were conducted using the PROCESS macro for SPSS version 22 (Hayes, 2013), with bootstrapping techniques (10,000 resamples) to determine 95% confidence intervals, as this macro is capable of calculating mediation effects for dichotomous outcome variables. Missing data were excluded pairwise from all the analyses. An alpha level of 0.05 was used for all analyses.

## Results

### Percentage of Eating Disorder Symptoms and ED Proneness Across Groups

The percentage of endorsed eating disorder behaviors and attitudes across groups is shown in Table 2. There was a significant difference in the percentage of ED proneness between groups, with lesbian women being more likely than gay men to have an ESP score of two or more (*OR*, 2.09; 95% CI, 1.08–4.05). There was low endorsement of the inappropriate weight control behaviors of taking diet pills or vomiting/taking laxatives to lose weight in the previous 30 days, with no significant difference between groups for diet pill use or vomiting/laxative use. Nor was there a significant difference between groups for eating in secret. However, there was a significant difference between groups in terms of weight-based self-worth, with lesbian women being more likely than gay men (*OR*, 2.52; 95% CI, 1.19–5.37) and TGNC adults (*OR*, 2.44; 95% CI, 1.17–5.09) to endorse this item. There was also a significant difference in self-reported current or past experience of an eating disorder, with lesbian women (*OR*, 2.83; 95% CI, 1.30–6.33) and TGNC adults (*OR*, 2.32; 95% CI, 1.10–4.88) being more likely than gay men to endorse this item. Finally, there was also a significant group difference for dissatisfaction with eating patterns, with the TGNC adults being more likely to be dissatisfied with their eating patterns than gay men (*OR*, 2.30; 95% CI, 1.27–4.19).

**Table 2:** Number (Percentage) of participants who endorsed eating disorder attitudes/behaviors and met the cut-off for ED proneness.

	**Gay men**		**Lesbian women**		**TGNC adults**	
	***n***	**Frequency**	***n***	**Frequency**	***n***	**Frequency**
ED proneness (ESP = 2+)	84	40 (47.6%)	72	48 (66.7%)^a^	115	72 (62.6%)
Eating in secret	86	20 (23.3%)	72	23 (31.9%)	115	26 (22.6%)
Weight affects the way they feel about themselves	84	53 (63.1%)	72	59 (81.9%)^b^	116	78 (67.2%)
Currently suffer with or have ever suffered from an eating disorder	86	12 (14.0%)	72	25 (34.7%)^a^	116	35 (30.2%)^a^
Dissatisfied with eating patterns	86	41 (47.7%)	72	45 (62.5%)	116	81 (69.8%)^a^
Used diet pills	83	2 (2.4%)	67	3 (4.5%)	118	3 (2.5%)
Vomited or took laxatives	83	1 (1.2%)	67	1 (1.5%)	118	3 (2.5%)

*TGNC, Transgender and Non-conforming; ESP, Eating Disorders Screen for Primary Care*.

a *Significantly different to gay men*.

b *Significantly different to gay men and TGNC adults*.

### Predictors of ED Proneness Across Groups

Scores on each of the predictor variables across groups are shown in Table 3. One-way between group ANOVAs showed that there were significant differences between groups on each of the variables at the *p* < 0.001 level. *Post-hoc* comparisons using the Tukey HSD test indicated that gay men had significantly lower anxiety and negative social exchange mean scores than both lesbian women and TGNC adults. TGNC adults had significantly higher mean scores than gay men and lesbian women for depression, perceived stigma, negative social exchange, and significantly lower mean self-compassion scores.

**Table 3:** Comparison of mean scores on the predictor variables between groups.

**Variable**	**Gay men**			**Lesbian women**			**TGNC adults**			***F*(df)**
	***N***	**Mean**	***SD***	***n***	**Mean**	***SD***	***n***	**Mean**	***SD***
Depression (PHQ-9)	84	5.98	5.02	68	8.18	6.25	114	11.71^a^	7.32	20.20 (2, 265)
Anxiety (GAD-7)	87	5.70^a^	5.22	69	8.26	5.96	119	9.95	5.63	14.53 (2, 272)
Negative social exchange (MHP-P)	82	7.66^a^	3.43	69	9.28	4.39	116	10.12^a^	4.04	9.33 (2, 264)
Thwarted belongingness (INQ)	84	22.24	11.98	65	22.66	11.06	108	30.99	12.75	15.74 (2, 254)
Perceived stigma (PSS)	79	27.35	7.81	64	26.75	7.25	105	31.52^a^	6.00	12.51 (2, 245)
Self-compassion (SCS)	81	37.96	10.08	59	36.59	9.89	104	31.60^a^	9.13	11.14 (2, 243)

*TGNC, Transgender and Non-conforming; PHQ-9, Patient Health Questionnaire Depression Scale; GAD-7, Generalized Anxiety Scale; MHP-P, Negative Social Exchange subscale of the Multidimensional Health Profile: Psychological Functioning; INQ, Thwarted belongingness subscale of the Interpersonal Needs Questionnaire; PSS, Perceived Stigma Scale; SCS, Self-Compassion Scale – Short Form*.

a *post-hoc comparison using the Tukey HSD test indicating significance at the p < 0.05 level*.

Logistic regressions were performed to investigate the predictors of ED proneness for each group, as shown in Table 4. The factors that were significantly associated with ED proneness in gay men were depression, perceived stigma, and (inversely) self-compassion. In lesbian women, only depression was significantly associated with ED proneness, although there was a trend for anxiety as a significant predictor at the *p* = 0.10 level. Within the TGNC group, only self-compassion was significantly, inversely associated with ED proneness.

**Table 4:** Logistic regression testing predictors of ED proneness across groups.

**Variable**	**Gay men (*****n*** **= 67)**			**Lesbian women (*****n*** **= 50)**			**TGNC adults (*****n*** **= 91)**		
	**B**	***OR***	**95% CI**	**B**	***OR***	**95% CI**	**B**	***OR***	**95% CI**
Depression (PHQ-9)	0.223*	1.25	[1.00, 1.56]	0.313*	1.37	[1.04, 1.79]	−0.011	0.99	[0.89, 1.10]
Anxiety (GAD-7)	−0.004	1.00	[0.83, 1.20]	−0.220	0.80	[0.63, 1.02]	0.023	1.02	[0.89, 1.17]
Self-compassion (SCS)	−0.098*	0.91	[0.83, 0.99]	−0.059	0.94	[0.86, 1.04]	−0.109**	0.90	[0.84, 0.96]
Negative social exchange (MHP-P)	0.032	1.03	[0.84, 1.28]	0.147	1.16	[0.92, 1.46]	−0.007	0.99	[0.87, 1.14]
Thwarted belongingness (INQ)	−0.051	0.95	[0.88, 1.03]	−0.032	0.97	[0.87, 1.07]	−0.007	0.99	[0.94, 1.04]
Perceived stigma (PSS)	0.109*	1.12	[1.00, 1.24]	−0.039	0.96	[0.87, 1.08]	0.031	1.03	[0.95, 1.12]
Constant	−0.002	1.00		2.52	12.45		3.05	21.03

OR, odds ratio; CI, confidence interval; TGNC, Transgender and Non-conforming; PHQ-9, Patient Health Questionnaire Depression Scale; GAD-7, Generalized Anxiety Scale; SCS, Self-Compassion Scale – Short Form; MHP-P, Negative Social Exchange subscale of the Multidimensional Health Profile: Psychological Functioning; INQ, Thwarted belongingness subscale of the Interpersonal Needs Questionnaire; PSS, Perceived Stigma Scale;

* *p < 0.05*.

** *p < 0.01*.

### Mediation Analyses to Examine the Interpersonal Theory of Eating Disorders (IPT-ED)

**Figure 1:**
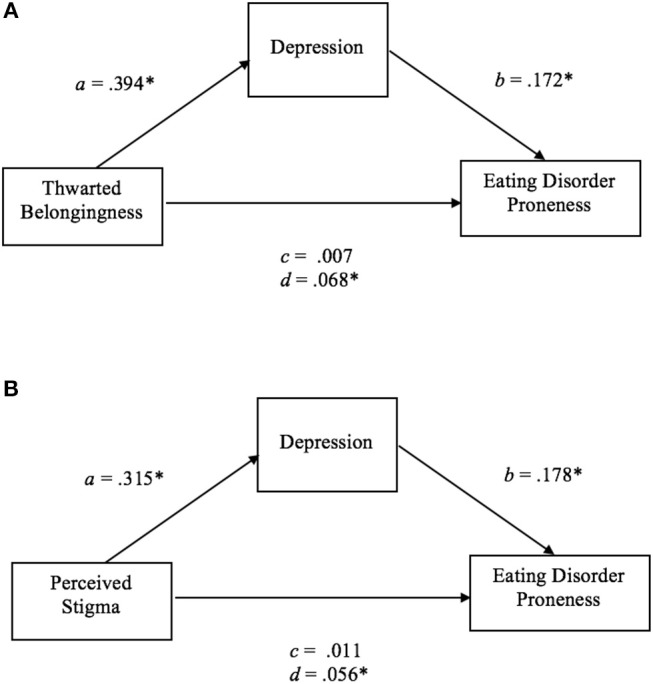
**(A)** Mediation model of thwarted belongingness on ED proneness in lesbian women. *a*, the effect of thwarted belongingness on depression; *b*, the effect of depression on ED proneness; *c*, the direct effect of thwarted belongingness on ED proneness; *d*, the indirect effect of thwarted belongingness on ED proneness with bootstrapping (10,000 resamples). *n* = 58. *p < 0.05. **(B)** Mediation model of perceived stigma on ED proneness in lesbian women*. a*, the effect of perceived stigma on depression; *b*, the effect of depression on ED proneness; *c*, the direct effect of perceived stigma on ED proneness; *d*, the indirect effect of perceived stigma on ED proneness with bootstrapping (10,000 resamples). *n* = 55. *p < 0.05.

**Figure 2:**
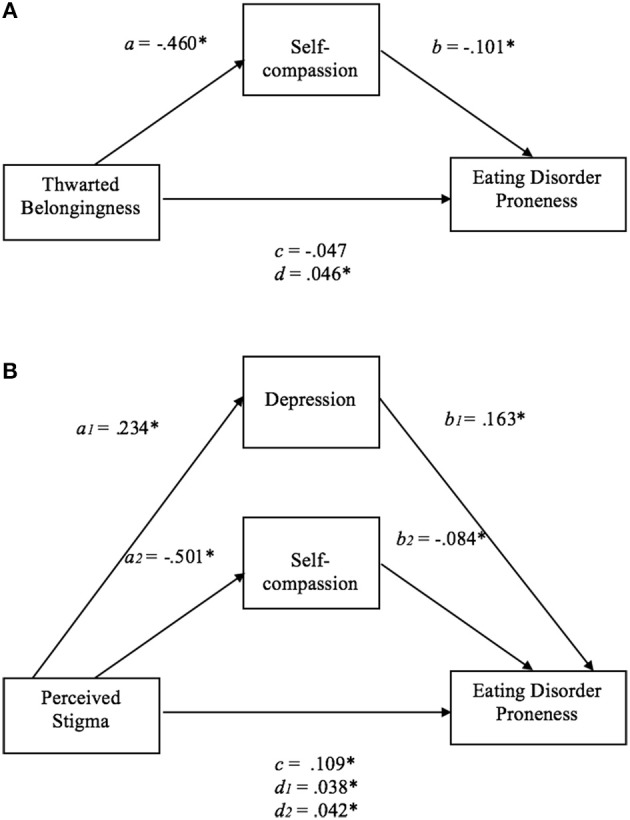
**(A)** Mediation model of thwarted belongingness on ED proneness in gay men. *a*, the effect of thwarted belongingness on self-compassion_;_ *b*, the effect of self-compassion on ED proneness; *c*, the direct effect of thwarted belongingness on ED proneness; *d*, the indirect effect of thwarted belongingness on ED proneness with bootstrapping (10,000 resamples). *n* = 74. *p < 0.05. **(B)** Mediation model of perceived stigma on ED proneness in gay men. *a*_1_, the effect of perceived stigma on depression;, *a*_2_, the effect of perceived stigma on self-compassion; *b*_1_, the effect of depression on ED proneness; *b*_2_, the effect of self-compassion on ED proneness; *c*, the direct effect of perceived stigma on ED proneness; *d*_1_, the indirect effect of perceived stigma on ED proneness with bootstrapping (10,000 resamples), mediated by depression; *d*_2_, the indirect effect of perceived stigma on ED proneness with bootstrapping (10,000 resamples), mediated by self-compassion. *n* = 71. *p < 0.05.

**Figure 3:**
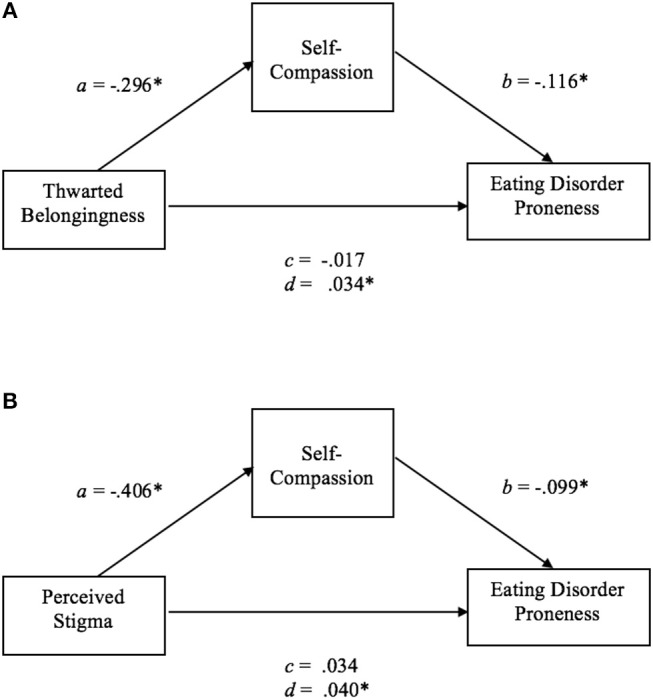
**(A)** Mediation model of thwarted belongingness on ED proneness in TGNC adults. *a*, the effect of thwarted belongingness on self-compassion; *b*, the effect of self-compassion on ED proneness; *c*, the direct effect of thwarted belongingness on ED proneness; *d*, the indirect effect of thwarted belongingness on ED proneness with bootstrapping (10,000 resamples). *n* = 96. *p < 0.05. **(B)** Mediation model of perceived stigma on ED proneness in TGNC adults. *a*, the effect of perceived stigma on self-compassion; *b*, the effect of self-compassion on ED proneness; *c*, the direct effect of perceived stigma on ED proneness; *d*, the indirect effect of perceived stigma on ED proneness with bootstrapping (10,000 resamples). *n* = 97. *p < 0.05.

Mediation analyses were performed to investigate whether the psychological factors that were significant predictor variables of ED proneness mediated the effect of interpersonal factors on ED proneness. Depression and self-compassion were significant predictor variables of ED proneness in the gay men, depression in the lesbian women, and self-compassion in the TGNC adults. As such, these psychological variables were included in the mediation analyses for each group. Thwarted belongingness, which had the largest correlations with depression and self-compassion in gay men (see Table 5), depression in lesbian women (see Table 6), and self-compassion in the TGNC group (see Table 7), was included as a representative interpersonal factor in the simple mediation analysis. As shown in the figures, there was a significant indirect effect between thwarted belongingness and ED proneness that was mediated by depression in lesbian women (Figure 1A) and lower self-compassion in gay men (Figure 2A) and TGNC adults (Figure 3A). Mediation analysis was also performed to examine whether perceived stigma as another interpersonal factor had an indirect relationship with ED proneness. As shown in the figures, there was a significant indirect effect between perceived stigma and ED proneness for each group. Perceived stigma was mediated by depression in lesbian women (Figure 1B), by depression and self-compassion in gay men (Figure 2B), and by self-compassion in TGNC adults (Figure 3B).

**Table 5:** Summary of intercorrelations between predictor variables for gay men.

	**Depression**	**Anxiety**	**Negative social exchange**	**Thwarted belongingness**	**Perceived stigma**	**Self compassion**
Depression	–	0.609**	0.560**	0.731**	0.342*	−0.522**
Anxiety		–	0.412**	0.427**	0.413**	−0.595**
Negative social exchange			–	0.494**	0.429**	−0.476**
Thwarted belongingness				–	0.360**	−0.551**
Perceived stigma					–	−0.383**
Self compassion						–

* *p < 0.01*.

** *p < 0.001*.

**Table 6:** Summary of intercorrelations between predictor variables for lesbian women.

	**Depression**	**Anxiety**	**Negative social exchange**	**Thwarted belongingness**	**Perceived stigma**	**Self compassion**
Depression	–	0.775***	0.483***	0.672***	0.388**	−0.456***
Anxiety		–	0.496***	0.617***	0.347***	−0.580***
Negative social exchange			–	0.501***	0.227	−0.278*
Thwarted belongingness				–	0.139	−0.449***
Perceived stigma					–	−0.406**
Self compassion						–

* *p < 0.05*.

** *p < 0.01*.

*** *p < 0.001*.

**Table 7:** Summary of intercorrelations between predictor variables for TGNC adults.

	**Depression**	**Anxiety**	**Negative social exchange**	**Thwarted belongingness**	**Perceived stigma**	**Self compassion**
Depression	–	0.763***	0.390***	0.549***	0.204*	−0.371***
Anxiety		–	0.447***	0.362***	0.317***	−0.337***
Negative social exchange			–	0.469***	0.201*	−0.208*
Thwarted belongingness				–	0.132	−0.424***
Perceived Stigma					–	−0.269**
Self compassion						—

* *p < 0.05*.

** *p < 0.001*.

*** *p < 0.001*.

## Discussion

In this study we aimed to build on the previous, albeit limited, research base to compare the relative levels of eating disorder symptoms and ED proneness between gay men, lesbian women, and a TGNC group. Moreover, we sought to identify and compare the risk and protective factors of ED proneness in these populations, and to examine the IPT-ED model, which posits that psychological factors related to the self and negative affect mediate the association between negative, socially-evaluative interpersonal factors and eating disorder symptoms.

In terms of rates of eating disorder symptoms, although previous literature has reported an elevated prevalence of inappropriate compensatory behaviors among sexual and gender minority populations (e.g., Diemer et al., 2015), this was less evident in the current sample where <3% of the total sample endorsed taking diet pills to lose weight, and <2% endorsed vomiting or taking laxatives to lose weight. The vast majority of prevalence data regarding eating disorder symptoms in sexual minority and gender diverse populations is derived from adolescents and young adult populations (Calzo et al., 2017). Given that the occurrence of maladaptive weight control behaviors decreases with age (Mond and Hay, 2007; Keel et al., 2010), the lower percentage of these behaviors in the present study may have been contributed to by age differences. For example, the median age of participants in the Diemer et al. study was 20 years, compared with the mean ages in the present study of 34 for the TGNC adults, 39 for the lesbian women, and 43 for the gay men.

A more frequent type of eating disorder pathology in the present sample was eating in secret, which was endorsed by 25.9% of the total sample. Even more common was weight-based self-worth, with lesbian women reporting the highest percentage. Although the literature regarding body image concerns in sexual minority and gender diverse women remains inconclusive (Bankoff and Pantalone, 2014), our finding that lesbian women had the highest percentage of weight-based self-worth is consistent with the conclusion of a meta-analysis by Morrison et al. (2004) that a lesbian sexual orientation may not necessarily protect women from body image concerns, as had been proposed by early theorists such as Siever (1994). In fact, lesbian women had a significantly higher percentage of ED proneness than the gay men (but were comparable in this regard to the TGNC group). This finding of higher ED proneness among the lesbian women is inconsistent with previous research suggesting comparable prevalence rates for eating disorders among gay men and lesbian women (Feldman and Meyer, 2007). The present finding that lesbian women had a higher percentage of ED proneness than gay men can be partly understood in terms of the ESP indexing *weight*-based self-worth and therefore being potentially insensitive to the muscularity-based concerns of men, with previous research supporting the notion of an elevated drive for muscularity within male sexual minority populations (Kimmel and Mahalik, 2005; Brennan et al., 2012).

From the findings reported in Table 2, it appears that ED proneness was largely contributed to by the ESP item, “Does your weight affect the way you feel about yourself?” This is not surprising given that weight- and/or shape-based self-worth is among the diagnostic features of most eating disorders (American College Health Association, 2014), longitudinally predicts the emergence of severe eating disorder behaviors (Neumark-Sztainer et al., 2007), and is considered to be the core cognitive dysfunction maintaining other eating disorder symptoms (Fairburn et al., 2003), rendering it a key target of treatment (Fairburn, 2008).

We hypothesized that the significant predictor variables of ED proneness would differ between the groups, especially among the TGNC adults for whom eating disordered attitudes and behaviors may be more the result of dissatisfaction with sex features that are inconsistent with one's gender identity than psychological disturbances such as negative affect. The results indicated that the predictor variables of ED proneness did indeed differ across groups. Negative mood was a predictor variable of ED proneness among gay men (depression) and lesbian women (depression and, marginally, anxiety). The role of negative affect for ED proneness among both gay men and lesbian women is consistent with the large body of theoretical (e.g., Stice, 2002; Fairburn et al., 2003; Rieger et al., 2010) and empirical (e.g., Stice et al., 2011; Hilbert et al., 2014) work implicating affective disturbance in the etiology and maintenance of eating disorders, with eating disordered behaviors potentially engaged in as a means of affect regulation. Mood disturbance was, in fact, the only predictor variable of ED proneness among the lesbian women, consistent with research indicating that problems such as emotional eating may be especially prominent among females (Tanofsky et al., 1997; Larsen et al., 2006). The fact that this prominent construct was not a predictor variable of ED proneness in the TGNC group provides some support for the notion that the etiology of eating disorder pathology in this population differs from the cisgender groups, and may be driven more so by dissatisfaction with sex features that are inconsistent with one's gender identity than general affective disturbance, resulting in these individuals seeking bodily modifications that are more consistent with their gender as suggested by Diemer et al. (2015) and Watson et al. (2017). Consequently, it cannot be assumed that variables have a comparable association with eating disorder pathology across populations of diverse gender and sexual orientations.

Results supported the hypothesis that perceived stigma would emerge as an additional risk factor for eating disorder susceptibility in this sexual minority and gender diverse sample. Perceived stigma was found to have a direct relationship with ED proneness for the gay men, and a significant indirect relationship with ED proneness in gay men, lesbian women, and TGNC adults. These findings provide further support for the deleterious effect that stigma has on mental health outcomes in sexual and gender minority populations (Hatzenbuehler and Pachankis, 2016) as well as being a risk factor for eating disorder symptoms and associated health risks within this population (Pachankis, 2015; Mason et al., 2017; Wang and Borders, 2017). Such findings are in accordance with the minority stress model, which highlights the role of stigma in the etiology of mental health problems among minority populations such as gay men and lesbian women (Meyer, 2003).

It was further expected that self-compassion might be especially relevant as a potential protective factor for ED proneness in this sexual minority and gender diverse sample, as it may function to protect the individual against the adverse consequences of stigmatizing environments. Partial support was found for this, in that lower self-compassion was a predictor variable of ED proneness in the gay men and TGNC adults. Evidence for the role of self-compassion is emerging (Braun et al., 2016), with the results of the present study supporting the need for further investigating the inclusion of self-compassion in existing models of eating disorders and therapeutic approaches. The results of the present study also support additional research on the role of self-compassion within sexual minority and gender diverse groups, and its interaction with stigma, in the development of theoretical and therapeutic approaches (Pachankis, 2015).

Despite higher levels of depression, anxiety, negative social exchange, thwarted belongingness, perceived stigma, and lower levels of self-compassion in the TGNC group compared to gay men and lesbian women, self-compassion was the only variable that demonstrated a unique association with ED proneness in the TGNC group. This finding again suggests that, in this population, eating disorder pathology is not primarily a means of affect regulation as proposed by theoretical models of eating disorders and, instead, provides further support for the notion that eating disorder symptoms among the TGNC population may be secondary to body dissatisfaction emerging from a body that is discrepant from one's gender identity (Jones et al., 2016; McClain and Peebles, 2016). That self-compassion was a significant predictor variable of ED proneness supports further investigation into the role of novel psychological interventions, such as Compassion-Focused Therapy, for supporting TGNC individuals, as has been recommended by Jones et al. (2016).

As well as seeking to understand some of the risk and protective factors relevant to eating disorder susceptibility in a sexual minority and gender diverse sample, the present study also sought to test the IPT-ED model (Rieger et al., 2010). Support was found for this approach. That is, while interpersonal factors did not have a direct association with ED proneness in any group (apart from perceived stigma in the gay men), thwarted belongingness and perceived stigma were found to have an indirect relationship that was mediated by lower self-compassion in the gay men and TGNC groups, and by depression among the lesbian women. The association between perceived stigma and ED proneness was also mediated by depression in the gay men. Thwarted belongingness and perceived stigma are manifestations of negative social evaluation, which is the core interpersonal factor deemed to be relevant for eating disorder symptoms within the IPT-ED model. According to this model, and supported by the findings, high levels of thwarted belongingness and perceived stigma would trigger disturbances of the self (i.e., lower self-compassion in the present study) and/or associated negative affect that, in turn, contribute to engagement in eating disorder pathology.

The present findings must be interpreted in the context of several methodological limitations. First, and most importantly, the ESP is a screening tool for eating disorders, with a sensitivity of 100% but a specificity of only 71%, as previously stated (Cotton et al., 2003). As such, while the ESP is helpful in identifying the vast majority, if not all, participants with an eating disorder, it also over-estimates the prevalence of eating disorders. Hence the present data should be interpreted as indexing susceptibility to an eating disorder rather than actual cases of eating disorders. Second, the sample sizes were relatively small and, therefore, only large effect sizes would likely have been detected (Agresti, 2007). Moreover, due to the small sample size of sub-groups within the TGNC group, this group was diverse and included those who identified as non-binary, intersex, trans-males, and trans-females, who may each experience different stressors and be vulnerable to different eating disorder symptoms (e.g., seeking to attain different body ideals) (Jones et al., 2016; McClain and Peebles, 2016). Third, future research on sexual minority and gender diverse groups would benefit from including additional key variables known to be related to eating disorder symptoms, such as perfectionism and low self-esteem. Of particular interest would be research examining potential moderating variables since not all individuals exposed to forms of negative social evaluation (such as stigma) experience eating disordered behaviors. One possible moderating variable proposed by Rieger et al. (2010) is a person's outcome expectancies, such as the belief that engaging in binge eating will help to reduce one's emotional distress or that social approval can be attained through thinness. Fourth, respondents self-selected to participate in a study advertised as investigating resilience and health-related quality of life in the LGBTQ community, and as such, the findings are subject to non-response bias (e.g., those without an interest in resilience and health-related quality of life in the LGBTQ community choosing not to participate) and thus may not reflect the broader sexual minority and gender diverse community. This may have contributed to the high percentage of ED proneness in the present study, with those individuals experiencing health-related problems such as eating disorder pathology potentially being more likely to participate. Finally, the study was cross-sectional in design, thereby precluding causal statements. Hence, prospective and experimental research is needed to substantiate the causal role of the predictor and mediator variables identified in the study.

In summary, this study contributes to an understanding of the relative levels of eating disorder symptoms among different sexual and gender minority populations, as well as the potential pathways to ED proneness among these groups. While the results of this study indicated low levels of maladaptive weight control behaviors (i.e., diet pill use and purging), there were high percentages of weight-based self-worth and ED proneness within our sample, especially among the lesbian women. There were also differences between groups regarding risk and protective factors of ED proneness, which support the notion that eating disorder pathology within TGNC adults might reflect dissatisfaction with undesired sex features that are discrepant from one's gender identity as opposed to traditional risk factors such as negative affect. The results provide evidence of the direct and indirect role of perceived stigma, a construct not currently included in theoretical models or intervention approaches, on ED proneness. Moreover, the finding that higher self-compassion in the face of negative interpersonal experiences (i.e., thwarted belongingness and stigma) was associated with lower ED proneness in gay men and TGNC adults supports further investigation into this construct within the field of eating disorders.

## Author Contributions

JH designed the larger study and provided access to ER and KB to design the present study. KB managed the data file and undertook the statistical analyses and took the lead in writing the manuscript. ER and JH provided input into successive drafts of the manuscript.

### Conflict of Interest Statement

The authors declare that the research was conducted in the absence of any commercial or financial relationships that could be construed as a potential conflict of interest.

## References

[B1] Agresti A. (2007). Logistic Regression: An Introduction to Categorical Data Analysis, 2nd Edn. Hoboken, NJ: John Wiley and Sons.

[B2] Almeida J. Johnson R. M. Corliss H. L. Molnar B. E. Azrael D. (2009). Emotional distress among LGBT youth: the influence of perceived discrimination based on sexual orientation. J. Youth Adol. 38, 1001–1014. 10.1007/s10964-009-9397-9 19636742 PMC3707280

[B3] American College Health Association (2014). National Health College Assessment. Available online at: http://www.acha-ncha.org/

[B4] American Psychiatric Association (2013). Diagnostic and Statistical Manual of Mental Disorders, 5th Edn. Arlington, VA: American Psychiatric Publishing.

[B5] American Psychiatric Association (2015). Guidelines for Psychological Practice With Transgender and Gender Non-conforming People. Available online at: http://www.apa.org/practice/guidelines/transgender.pdf

[B6] Austin S. B. Nelson L. A. Birkett M. A. Calzo J. P. Everett B. (2013). Eating disorder symptoms and obesity at the intersections of gender, ethnicity, and sexual orientation in US high school students. Am. J. Public Health 103, e16–e22. 10.2105/AJPH.2012.301150 23237207 PMC3558764

[B7] Bankoff S. M. Pantalone D. W. (2014). Patterns of disordered eating behavior in women by sexual orientation: a review of the literature. Eating Disord. 22, 261–274. 10.1080/10640266.2014.890458 24617312

[B8] Bayer V. Robert-McComb J. J. Clopton J. R. Reich D. A. (2017). Investigating the influence of shame, depression, and distress tolerance on the relationship between internalized homophobia and binge eating in lesbian and bisexual women. Eating Behav. 24, 39–44. 10.1016/j.eatbeh.2016.12.001 27992763

[B9] Beard K. Eames C. Withers P. (2017). The role of self-compassion in the well-being of self-identifying gay men. J. Gay Lesb. Mental Health 21, 77–96. 10.1080/19359705.2016.1233163

[B10] Braun T. D. Park C. L. Gorin A. (2016). Self-compassion, body image, and disordered eating: a review of the literature. Body Image 17, 117–131. 10.1016/j.bodyim.2016.03.003 27038782

[B11] Brennan D. J. Craig S. L. Thompson D. E. A. (2012). Factors associated with a drive for muscularity among gay and bisexual men. Cult. Health Sex. 14, 1–15. 10.1080/13691058.2011.619578 22077494

[B12] Calzo J. P. Blashill A. J. Brown T. A. Argenal R. L. (2017). Eating disorders and disordered weight and shape control behaviors in sexual minority populations. Curr. Psychiatry Rep. 19:49. 10.1007/s11920-017-0801-y 28660475 PMC5555626

[B13] Cotton M. Ball C. Robinson P. (2003). Four simple questions can help screen for eating disorders. J. General Inter. Med. 1, 53–56. 10.1046/j.1525-1497.2003.20374.x PMC1494802 12534764

[B14] Diemer E. W. Grant J. D. Munn-Chernoff M. A. Patterson D. A. Duncan A. E. (2015). Gender identity, sexual orientation, and eating-related pathology in a national sample of college students. J. Adol. Health 57, 144–149. 10.1016/j.jadohealth.2015.03.003 25937471 PMC4545276

[B15] Fairburn C. G. (2008). Cognitive Behavior Therapy and Eating Disorders. New York, NY: Guildford Press.

[B16] Fairburn C. G. Cooper Z. Shafran R. (2003). Cognitive behaviour therapy for eating disorders: a “transdiagnostic” theory and treatment. Behav. Res. Ther. 41, 509–528. 10.1016/S0005-7967(02)00088-8 12711261

[B17] Feldman M. B. Meyer I. H. (2007). Eating disorders in diverse lesbian, gay, and bisexual populations. Int. J. Eat. Disord. 40, 218–226. 10.1002/eat.20360 17262818 PMC2080655

[B18] Feldman M. B. Meyer I. H. (2010). Comorbidity and age of onset of eating disorders in gay men, lesbians, and bisexuals. Psychiatry Res. 180, 126–131. 10.1016/jpsychres.2009.10.013 20483473 PMC3726047

[B19] Geller J. Srikameswaran S. Zelichowska J. (2015). Resilience to shape and weight concerns and disordered eating: the role of self-compassion. Adv. Eating Disord. 3, 4–12. 10.1080/21662630.2014.945604

[B20] Hatzenbuehler M. L. Pachankis J. E. (2016). Stigma and minority stress as social determinants of health among lesbian, gay, bisexual, and transgender youth: research evidence and clinical implications. Pediatr. Clin. North Am. 63, 985–997. 10.1016/j.pcl.2016.07.003 27865340

[B21] Hayes A. F. (2013). Introduction to Mediation, Moderation, and Conditional Process Analysis: A Regression-Based Approach. New York, NY: Guildford Press.

[B22] Herr N. R. Williams J. W. Jr. Benjamin S. McDuffie J. (2014). Does this patient have generalized anxiety or panic disorder? The Rational Clinical Examination systematic review. J. Am. Med. Assoc. 312, 78–84. 10.1001/jama.2014.5950 25058220

[B23] Hilbert A. Pike K. Goldschmidt A. B. Wilfley D. E. Fairburn C. G. Dohm F.. (2014). Risk factors across eating disorders. Psychiatry Res. 220, 500–506. 10.1016/j.psychres.2014.05.054 25103674 PMC4785871

[B24] Hirsch J. Kaniuka A. Brooks B. Hirsch K. K. Mann A. Williams S. L. (2017). What the trump? Anticipated rejection and concern about rights are associated with suicide risk in LGBTQ communities but can resilience trump risk? Clin. Psychol. 70, 17–20.

[B25] Jones B. A. Haycraft E. Murjan S. Arcelus J. (2016). Body dissatisfaction and disordered eating in trans people: a systematic review of the literature. Int. Rev. Psychiatry 28, 81–94. 10.3109/09540261.2015.1089217 26618239

[B26] Keel P. K. Gravener J. A. Joiner T. E. Haedt A. A. (2010). Twenty-year follow-up of bulimia nervosa and related eating disorders not otherwise specified. Int. J. Eat. Disord. 43, 492–497. 10.1002/eat.20743 19718666

[B27] Kelleher C. (2009). Minority stress and health: implications for lesbian, gay, bisexual, transgender, and questioning (LGBTQ) young people. Counsell. Psychol. Q. 22, 373–379. 10.1080/09515070903334995

[B28] Kelly A. C. Tasca G. A. (2016). Within-persons predictors of change during eating disorders treatment: an examination of self-compassion, self-criticism, shame, and eating disorder symptoms. Int. J. Eat. Disord. 49, 716–722. 10.1002/eat.22527 27061929

[B29] Keng S.-L. Liew K. W. L. (2017). Trait mindfulness and self-compassion as moderators of the association between gender non-conformity and psychological health. Mindfulness 8, 615–626. 10.1007/s12671-016-0639-0

[B30] Kimmel S. B. Mahalik J. R. (2005). Body image concerns of gay men: The roles of minority stress and conformity to masculine norms. J. Consult. Clin. Psychol. 73, 1185–1190. 10.1037/0022-006X.73.6.1185 16392992

[B31] Kroenke K. Spitzer R. L. Williams J. B. (2001). The PHQ-9: validity of a brief depression severity measure. J. General Int. Med. 16, 606–613. 10.1046/j.1525-1497.2001.016009606.x 11556941 PMC1495268

[B32] Larsen J. K. van Strien T. Eisinga R. Engels R. C. M. E. (2006). Gender differences in the association between alexithymia and emotional eating in obese individuals. J. Psychosomat. Res. 60, 237–234. 10.1016/j.jpsychores.2005.07.006 16516654

[B33] Lewis R. J. Derlega V. J. Griffin J. L. Krowinski A. C. (2003). Stressors for gay men and lesbians: life stress, gay-related stress, stigma consciousness, and depressive symptoms. J. Soc. Clin. Psychol. 22, 716–729. 10.1521/jscp.22.6.716.22932

[B34] MacBeth A. Gumley A. (2012). Exploring compassion: a meta-analysis of the association between self-compassion and psychopathology. Clin. Psychol. Rev. 32, 545–552. 10.1016/j.cpr.2012.06.003 22796446

[B35] Martin A. Rief W. Klaiberg A. Braehler E. (2006). Validity of the brief Patient Health Questionnaire mood scale (PHQ-9) in the general population. Gen. Hosp. Psychiatry 28, 71–77. 10.1016/j.genhosppsych.2005.07.003 16377369

[B36] Mason T. B. Lewis R. J. Heron K. E. (2017). Indirect pathways connecting sexual orientation and weight discrimination to disordered eating among young adult lesbians. Psychol. Sex. Orient. Gender Diver. 4, 193–204. 10.1037/sgd0000220

[B37] McClain Z. Peebles R. (2016). Body image and eating disorders among lesbian, gay, bisexual, and transgender youth. Pediatr. Clin. North Am. 63, 1079–1090. 10.1016/j.pcl.2016.07.008 27865334 PMC6402566

[B38] Meyer I. H. (2003). Prejudice, social stress, and mental health in lesbian, gay, and bisexual populations: conceptual issues and research evidence. Psychol. Bull. 129, 674–697. 10.1037/0033-2909.129.5.674 12956539 PMC2072932

[B39] Mickelson K. D. (2001). Perceived stigma, social support, and depression. Personal. Soc. Psychol. Bull. 27, 1046–1056. 10.1177/0146167201278011

[B40] Mond J. M. Hay P. J. (2007). Functional impairment associated with bulimic behaviors in a community sample of men and women. Int. J. Eating Disord. 40, 391–398. 10.1002/eat.20380 17497705

[B41] Morrison M. A. Morrison T. G. Sager C. L. (2004). Does body satisfaction differ between gay men and lesbian women and heterosexual men and women? a meta-analytic review. Body Image 1, 127–138. 10.1016/j.bodyim.2004.01.002 18089146

[B42] Neff K. (2003). Self-compassion: an alternative conceptualization of a healthy attitude toward oneself. Self Identity 2, 85–101. 10.1080/15298860309032

[B43] Neumark-Sztainer D. R. Wall M. M. Haines J. I. Story M. T. Sherwood N. E. van den Berg P. A. (2007). Shared risk and protective factors for overweight and disordered eating in adolescents. Am. J. Prevent. Med. 33, 359–369. 10.1016/j.amepre.2007.07.031 17950400

[B44] Pachankis J. E. (2015). A transdiagnostic minority stress treatment approach for gay and bisexual men's syndemic health conditions. Arch. Sex. Behav. 44, 1843–1860. 10.1007/s10508-015-0480-x 26123065 PMC4560958

[B45] Pennesi J. Wade T. (2016). A systematic review of the existing models of disordered eating: do they inform the development of effective interventions? Clin. Psychol. Rev. 43, 175–192. 10.1016/j.cpr.2015.12.004 26781985

[B46] Raes F. Pommier E. Neff K. D. Van Gucht D. (2011). Construction and factorial validation of a short form of the self-compassion scale. Clin. Psychol. Psychother. 18, 250–255. 10.1002/cpp.702 21584907

[B47] Rieger E. Van Buren D. Bishop M. Tanofsky-Kraff M. Welch R. Wilfley D. (2010). An eating disorder specific model of interpersonal psychotherapy (IPT-ED): causal pathways and treatment implications. Clin. Psychol. Rev. 30, 400–410. 10.1016/j.cpr.2010.02.001 20227151

[B48] Ruehlman L. S. Lanyon R. I. Karoly P. (1999). Development and validation of the multidimensional health profile, part 1: psychosocial functioning. Psychol. Assessm. 11, 166–176. 10.1037/1040-3590.11.2.166 PMC1317088 16429599

[B49] Siever M. D. (1994). Sexual orientation and gender as factors in socioculturally acquired vulnerability to body dissatisfaction and eating disorders. J. Consult. Clin. Psychol. 62, 252–260. 8201061 10.1037//0022-006x.62.2.252

[B50] Silva C. Ribeiro J. D. Joiner T. E. (2015). Mental disorders and thwarted belongingness, perceived burdensomeness, and acquired capability for suicide. Psychiatry Res. 226, 316–327. 10.1016/j.psychres.2015.01.008 25650048

[B51] Smink F. R. E. van Hoeken D. Hoek H. W. (2013). Epidemiology, course, and outcome of eating disorders. Curr. Opin. Psychiatry 26, 543–548. 10.1097/YCO.0b013e328365a24f 24060914

[B52] Spitzer R. L. Kroenke K. Williams J. B. W. Lowe B. (2006). A brief measure for assessing generalized anxiety disorder. Arch. Int. Med. 166, 1092–1097. 10.1001/archinte.166.10.1092 16717171

[B53] Steinhausen H. (2002). The outcome of anorexia nervosa in the 20th century. Am. J. Psychiatry 159, 1284–1293. 10.1176/appi.ajp.159.8.1284 12153817

[B54] Stice E. (2002). Risk and maintenance factors for eating pathology: a meta-analysis review. Psychol. Bull. 128, 825–848. 10.1037//0033-2909.128.5.825 12206196

[B55] Stice E. Marti C. N. Durant S. (2011). Risk factors for onset of eating disorders: Evidence of multiple risk pathways from an 8-year prospective study. Behav. Res. Therapy 49, 622–627. 10.1016/j.brat.2011.06.009 21764035 PMC4007152

[B56] Striegel-Moore R. H. Bulik C. M. (2007). Risk factors for eating disorders. Am. Psychol. 62, 181–198. 10.1037/0003-066X.62.3.181 17469897

[B57] Tanofsky M. B. Wilfley D. E. Spurrell E. B. Welch R. Brownell K. D. (1997). Comparison of men and women with binge eating disorder. Int. J. Eating Disord. 21, 49–54. 8986517 10.1002/(sici)1098-108x(199701)21:1<49::aid-eat6>3.0.co;2-3

[B58] Tylka T. L. Russell H. L. Neal A. A. (2015). Self-compassion as a moderator of thinness-related pressure' associations with thin-ideal internalization and disordered eating. Eating Behav. 17, 23–26. 10.1016/j.eatbeh.2014.12.009 25536526

[B59] Van Orden K. A. Cokrowicz K. C. Witte T. K. Joiner T. E. Jr. (2012). Thwarted belongingness and perceived burdensomeness: construct validity and psychometric properties of the interpersonal needs questionnaire. Psychol. Assessm. 24, 197–215. 10.1037/a0025358 21928908 PMC3377972

[B60] van Son G. E. van Hoeken D. van Furth E. F. Donker G. A. Hoek H. W. (2010). Course and outcome of eating disorders in a primary care-based cohort. In. J. Eating Disord. 43, 130–138. 10.1002/eat.20676 19308996

[B61] Vigna A. J. Poehlmann-Tynan J. Koenig B. W. (2017). Does self-compassion facilitate resilience to stigma? A school-based study of sexual and gender minority youth. Mindfulness 9, 914–924. 10.1007/s12671-017-0831-x

[B62] Wade T. D. Wilksch S. M. Lee C. (2012). A longitudinal investigation of the impact of disordered eating on young women's quality of life. Health Psychol. 31, 352–359. 10.1037/a0025956 22059619

[B63] Wang S. B. Borders A. (2017). Rumination mediates the associations between sexual minority stressors and disordered eating, particularly for men. Eating Weight Disord. 22, 699–706. 10.1007/s40519-016-0350-0 28039668

[B64] Watson R. J. Veale J. F. Seawyc E. M. (2017). Disordered eating behaviors among transgender youth: probability profiles from risk and protective factors. Int. J. Eating Disord. 50, 515–522. 10.1002/eat.22627 27862124 PMC5754211

