# Corrigendum: Eating Disorder Symptoms and Proneness in Gay Men, Lesbian Women, and Transgender and Gender Non-conforming Adults: Comparative Levels and a Proposed Mediational Model

**DOI:** 10.3389/fpsyg.2019.01540

**Published:** 2019-07-10

**Authors:** Kathryn Bell, Elizabeth Rieger, Jameson K. Hirsch

**Affiliations:** ^1^Research School of Psychology, Australian National University, Canberra, ACT, Australia; ^2^Department of Psychology, East Tennessee State University, Johnson City, TN, United States

**Keywords:** eating disorders, sexual minority and gender diverse, self-compassion, stigma, interpersonal, LGBTQ

In the original article, there was an error. The article referred to “transgender and non-conforming” people and should instead have referred to “transgender and gender non-conforming” people.

A correction has been made to the ***Title***:

“Eating Disorder Symptoms and Proneness in Gay Men, Lesbian Women, and Transgender and Gender Non-conforming Adults: Comparative Levels and a Proposed Mediational Model.”

A correction has also been made to the ***Abstract***.

“In this study we sought to compare eating disorder attitudes and behaviors, and proneness to an eating disorder (“ED proneness”), between gay men, lesbian women, and transgender and gender non-conforming (TGNC) adults. A further aim was to identify and compare risk and protective factors, and examine a mediational model based on the interpersonal theory of eating disorders (IPT-ED), whereby the association between interpersonal factors and ED proneness would be mediated by psychological constructs pertaining to the self and negative affect. Data was obtained from a larger national study of health risk and protective factors among sexual minority and gender diverse populations. The sample included 97 gay men, 82 lesbian women, and 138 TGNC adults. Participants completed the National College Health Assessment, Eating Disorders Screen for Primary Care, Patient Health Questionnaire Depression scale, Generalized Anxiety Disorder 7 scale, Self-Compassion Scale-Short Form, Negative Social Exchange subscale of the Multidimensional Health Profile, Interpersonal Needs Questionnaire, and Perceived Stigma Scale. There was a significant difference between groups in ED proneness, with lesbian women (66.7%) having a significantly higher percentage than gay men (47.6%). There was also a significant difference between groups in weight-based self-worth, with the lowest percentage in gay men (63%) and the highest percentage in lesbian women (82%), as well as dissatisfaction with eating patterns, with the highest percentage in TGNC adults (69.8%) and the lowest percentage in gay men (47.7%). There was a low percentage of inappropriate compensatory behaviors, with no significant difference between groups. Logistic regression analyses showed that the predictor variables of ED proneness were depression, perceived stigma, and self-compassion in gay men; depression in lesbian women; and self-compassion in the TGNC adults. Mediation analyses showed that thwarted belongingness (i.e., an unmet to belong) and perceived stigma had an indirect association with ED proneness that was mediated by self-compassion and depression (for perceived stigma alone) in gay men, depression in lesbian women, and self-compassion in TGNC adults. The interpersonal theory of eating disorders therefore extends to sexual minority and gender diverse populations; however, the results suggest a broadening of theoretical models and intervention programs to include the role of stigma and self-compassion.”

Lastly, a correction has been made to the ***Introduction***, paragraph five:

“Even less research has been conducted on transgender and gender non-conforming (TGNC) people (American Psychiatric Association, 2015). A large-scale study of college students by Diemer et al. (2015) found that the prevalence of self-reported disordered eating and inappropriate compensatory behavior was higher among transgender students than cisgender gay or heterosexual students. McClain and Peebles (2016) explain these findings by noting that some TGNC people experience dissatisfaction or distress in relation to features of their biological sex that are inconsistent with their gender identity, and propose that for these individuals the risk of eating disorder symptoms may be secondary to this body dissatisfaction or distress. For example, extreme weight loss strategies among TGNC people may be a means to inhibit undesired, or develop desired, gender features (Diemer et al., 2015; Watson et al., 2017). If this is the case, eating disorder symptoms in this group might be best alleviated with strategies to ameliorate this body dissatisfaction, including social transition, puberty blockers, cross-sex hormones, and gender affirmation surgery compared to implementing standard treatment approaches for eating disorders. In accordance with this suggestion, a systematic review of body dissatisfaction and disordered eating in TGNC people by Jones et al. (2016) found that body image improved after hormone or surgical treatments targeting dissatisfaction with the physical characteristics of one's biological sex that were discrepant from one's gender identity.”

Additionally, there was an error in the ***Materials and Methods***. The number of gay men should be “98” and the number of lesbian women should be “84.”

A correction has been made to the ***Materials and Methods***, subsection ***Participants***, paragraph two:

“In the current study, those who identified their birth sex and gender identity as male, and sexual orientation as gay were grouped as “gay men.” Those who identified their birth sex and gender identity as female, and sexual orientation as lesbian were grouped as “lesbian women.” Participants who indicated that their gender identity was different from their birth sex (*n* = 26) or who identified as transgender male to female (*n* = 14), transgender female to male (*n* = 21), gender queer (*n* = 21), gender-fluid (*n* = 8), non-binary (*n* = 17), agender (*n* = 6), two-spirit (*n* = 5), intersex (*n* = 2), or other (*n* = 18) were collectively grouped as “TGNC” so as to maximize power. The final sample was comprised of 98 gay men (mean age = 42.54, *SD* = 17.19; 74.5% Caucasian), 84 lesbian women (mean age = 38.64, *SD* = 17.65; 84.5% Caucasian), and 138 TGNC adults (mean age = 33.60, *SD* = 16.80; 83.3% Caucasian). Within the TGNC group, 46 identified their sex assigned at birth as male, 89 as female, and three declined to answer. Demographic characteristics of the three groups are shown in Table 1.”

**Table 1:** Participant demographic characteristics in percentages.

	**Gay men (*n* = 98)**	**Lesbian women (*n* = 84)**	**TGNC adults (*n* = 138)**
Mean Age (SD)	42.54 (17.19)	38.64 (17.65)	33.60 (16.80)
**LEGAL MARITAL STATUS**			
Single, never married	49.0	50.0	48.6
Married	20.4	21.4	21.7
Divorced	7.1	2.4	21.7
Widowed	1.0	0.0	0.0
Partnered, not legally married	15.3	21.4	16.7
Other	4.1	4.8	1.4
Decline to answer	1.0	0.0	1.4
Missing response	2.0	0.0	0.0
**HIGHEST EDUCATION LEVEL**			
Some high school	0.0	0.0	3.6
High school (includes GED)	4.1	3.6	3.6
Some college (no degree)	19.4	26.2	34.1
Associate's degree (2 years of college)	3.1	10.7	7.2
Bachelor degree (4 years of college)	33.7	21.4	23.9
Masters degree	27.6	23.8	19.6
Doctoral degree	12.2	14.3	7.2
Missing response	0.0	0.0	0.7
**EMPLOYMENT STATUS**			
Full time, paid	54.1	41.7	40.6
Part time, paid	5.1	10.7	12.3
Student	15.3	28.6	26.1
Homemaker	0.0	0.0	0.7
Retired	19.4	6.0	4.3
On disability	0.0	6.0	3.6
Unemployed, seeking paid employment	1.0	0.0	4.3
Unemployed, not seeking paid employment	0.0	1.2	0.7
Other	5.1	6.0	7.2
**ETHNICITY**			
White/Caucasian	74.5	84.5	83.3
Black or African American	2.0	1.2	2.2
Hispanic or Latino/a	9.2	0.0	1.4
American Indian or Alaska Native	0.0	1.2	0.7
Asian (including Asian Indian)	4.1	3.6	0.7
Multiracial	3.1	7.1	5.8
Other	7.1	0.0	4.3
Decline to answer	0.0	0.0	1.4
Missing data	0.0	1.2	0.0

Lastly, an error was made in Table 1 as published. The mean age of lesbian women should be “38.64.” The corrected Table 1 appears below.

The authors apologize for these errors and state that they do not change the scientific conclusions of the article in any way. The original article has been updated.
